# Erratum

**Published:** 2014-11-07

## Vol. 63, No. Suppl 4

In the *MMWR* Supplement, “CDC National Health Report: Leading Causes of Morbidity and Mortality and Associated Behavioral Risk and Protective Factors—United States, 2005–2013,” an error occurred in Table 4 on page 25. Three rates for 2013 for foodborne illness (0.26 for *Listeria* infection, 15.19 for *Salmonella* infection, and 1.15 for Shiga toxin–producing *Escherichia coli* infection) should have been footnoted as “data are preliminary.”

